# Efficient Selection Scheme for Incorporating Noncanonical Amino Acids Into Proteins in *Saccharomyces cerevisiae*

**DOI:** 10.3389/fbioe.2020.569191

**Published:** 2020-09-15

**Authors:** Linzhi Tan, Zhaohui Zheng, Yuanwei Xu, Weikaixin Kong, Zhen Dai, Xuewen Qin, Tao Liu, Hongting Tang

**Affiliations:** ^1^Center for Synthetic Biochemistry, Shenzhen Institutes for Advanced Technologies, Chinese Academy of Sciences, Shenzhen, China; ^2^State Key Laboratory of Natural and Biomimetic Drugs, Department of Molecular and Cellular Pharmacology, School of Pharmaceutical Sciences, Peking University, Beijing, China

**Keywords:** expanded genetic code, non-canonical amino acid, protein engineering, cell factory, *Saccharomyces cerevisiae*

## Abstract

With the advances in the field of expanded genetic code, the application of non-canonical amino acid (ncAA) is considered an effective strategy for protein engineering. However, cumbersome and complicated selection schemes limit the extensive application of this technology in *Saccharomyces cerevisiae*. To address this issue, a simplified selection scheme with confident results was developed and tested in this study. Based on a mutation library derived from *Escherichia coli* tyrosyl-tRNA synthetase (*Ec*TyrRS), a logic gate in synthetic biology was used to optimize screening procedures. We found that an “and” gate was more suitable than an “or” gate for isolating aminoacyl-tRNA synthetase from *S. cerevisiae*. The successful incorporation of *O*-methyltyrosine (OMeY) proved the utility and efficiency of this new selection scheme. After a round of positive selection, several new OMeY-tRNA synthetase (OMeYRS) mutants were screened, and their incorporation efficiency was improved. Furthermore, we characterized the insertion of several tyrosine analogs into Herceptine Fab and discovered that OMeYRS and its mutants were polyspecific. One of these mutants showed an optimal performance to incorporate different ncAAs into recombinant proteins in *S. cerevisiae*; this mutant was cloned and transfected into mammalian cells, and the results proved its functionality in HEK293 cells. This study could expand the application of ncAA in *S. cerevisiae* to construct efficient yeast cell factories for producing natural and synthetic products.

## Introduction

Most proteins are built from 20 proteinogenic amino acids. For decades, scientists have focused on modifying proteins with ncAA via solid-phase peptide synthesis (Merrifield, 1969), expressed protein ligation (Ambrogelly et al., 2007), cell-free translation (Martin et al., 2018), and *in vivo* expression using genetic code expansion (Liu and Schultz, 2010). Genetic code expansion enables the incorporation of ncAA into target proteins at specific sites upon recognition of nonsense codons by orthogonal aminoacyl-tRNA synthetase and its cognate tRNA (aaRS/tRNA) in living cells. Proteins with ncAA may acquire new physicochemical, biological, and pharmacological properties. Furthermore, incorporated ncAA can be used as probes to investigate protein structure or function (Liu and Schultz, 2010).

To ensure the orthogonality and fidelity, engineered aaRS must aminoacylate its cognate tRNA with the corresponding ncAA, and no endogenous tRNA or native amino acid must be recognized. Moreover, the evolved orthogonal tRNA should not be recognized by endogenous aminoacyl-tRNA synthetases carrying native amino acids (Chatterjee et al., 2012; Cervettini et al., 2020). Several orthogonal aaRS/tRNA pairs have been developed, such as *Methanococcus janaschii* tyrosyl-tRNA synthetase (*Mj*TyrRS/tRNA^CUA^), *Escherichia coli* tyrosyl-tRNA synthetase (*Ec*TyrRS/tRNA^CUA^), and pyrrolysyl-tRNA synthetase (PylRS/tRNA^CUA^) from certain *Methanosarcina*. PylRS/tRNA^CUA^ enables the incorporation of lysine analogs in prokaryotes and eukaryotes. However, *Mj*TyrRS/tRNA^CUA^ is orthogonal in prokaryotes such as *E. coli* but not in eukaryotes, whereas *Ec*TyrRS/tRNA^CUA^ is orthogonal in eukaryotes rather than in prokaryotes (Chin, 2014). Even though numerous ncAAs of tyrosine analogs have been efficiently encoded in *E. coli*, only a limited set of structurally simple ncAAs can be inserted in *Saccharomyces cerevisiae* and mammalian cells. Thus, the development of an efficient evolution system for *Ec*TyrRS engineering is important.

The translation machinery of *S. cerevisiae* is highly homologous to that from other eukaryotes, including mammalian cells. Besides, due to features such as a well-defined genetic background, mature genetic manipulation, easiness to cultivate, *S. cerevisiae* is suitable for studying eukaryotic evolution of orthogonal *Ec*TyrRS/tRNA^CUA^ encoding tyrosine analogs. Site-saturated mutagenesis of *Ec*TyrRS and general selection schemes have been developed in *S. cerevisiae.* Using this method, several specific *Ec*TyrRS mutants have been successfully “cut and pasted” into mammalian cells to incorporate ncAA into proteins (Liu et al., 2007; Chatterjee et al., 2013). To establish the selection scheme, transcription factor Gal4p was used as an expression regulator of three reporter genes, *URA3*, *HIS3* and *LacZ*. Amber stop codons were introduced at two permissive sites of Gal4p. The *URA3* and *HIS3* were used for positive selection involving the suppression of the auxotrophic phenotype. The protein encoded by the *HIS3* gene is related to cell tolerance to 3-aminotriazole (3-AT) in a dose-dependent manner. Moreover, the expression of LacZ turns colonies blue in the presence of X-gal, a chromogenic substrate for β-galactosidase (Chin et al., 2003b). In addition, the expression of *URA3* leads to cell death in the presence of 5-fluoroorotic acid (5-FOA), since Ura3p converts 5-FOA into a toxic product. Thus, *URA3*/5-FOA was used as a negative selection system. In the first round of selection, cells were cultivated in the presence of target ncAA without uracil (Ura) or histidine (His) to screen active aaRS that can recognize read-through amber codons with ncAA or canonical amino acids. Surviving cells were grown in the presence of 5-FOA without ncAA to remove aaRS that could incorporate canonical amino acids. In general, three cycles of positive and negative selections yield specific aaRS that incorporate target ncAA into proteins (Chin et al., 2003a; Cropp et al., 2007). Although this selection scheme is efficient, it remains cumbersome and time-consuming (Grasso et al., 2019).

*S. cerevisiae* is widely used for producing bioproducts, addressing biological processes and displaying libraries of surface antibodies or enzymes (Nevoigt, 2008; Cherf and Cochran, 2015). The application of ncAA in *S. cerevisiae* is limited, due to the inefficiency of current orthogonal pairs. It was reported that the incorporation efficiency of ncAA increases significantly in *S. cerevisiae* upon optimization of tRNA expression cassette and interception of nonsense-mediated mRNA decay pathway, which plays an essential role in the rapid degradation of mRNA containing premature stop codons (Wang and Wang, 2008). However, optimization of aaRS efficiency is still relatively rare in *S. cerevisiae*. In addition, some aaRSs are polyspecific to several ncAAs in bacterial and mammalian cells (Young et al., 2011; Chatterjee et al., 2013). Thus, the availability of an efficient aaRS with polyspecificity is attractive in *S. cerevisiae* because a single construct could be used to incorporate different ncAAs.

In this study, a selection scheme of specific aaRS was optimized using the *Ec*TyrRS mutation library to incorporate OMeY. The traditional three-cycles of positive and negative selection was simplified to one round of positive selection. As a result, five novel OMeYRS mutants were selected and their charging efficiency was determined. In addition, the incorporation efficiency of several tyrosine analogs including *O*-Propargyl-L-tyrosine (OPrY), *O*-Bromoethyl-L-tyrosine (BetY), and *p*-2′-fluoroacetyl-L-phenylalanine (FpAcF) into Herceptine Fab was compared with that from OMeYRS mutants. One OMeYRS mutant was cloned and transfected into mammalian cells, and the results demonstrated that this mutant works in HEK293 cells.

## Materials and Methods

### Chemical Synthesis of Non-canonical Amino Acids

Solvents and reagents used for chemical synthesis were purchased from Sigma-Aldrich, TCI and Bide Pharmatech. The 0.25 mm silica gel 60-F254 was used for analytical thin layer chromatography, using UV light treatment and vanillin or ninhydrin staining. Flash column chromatography was carried out on silica gel (particle size 200–300 mesh, purchased from Qingdao Haiyang Chemical Co.). ^1^H and ^13^C NMR spectra were analyzed using Bruker AVANCE III-400 spectrometers. All ncAAs were synthesized as previously described (Tang et al., 2018).

### Strains and Media

*Escherichia coli* Trans5α was used for plasmid construction and propagation. Recombinant *E. coli* strains were grown in LB medium (5 g/L yeast extract, 10 g/L peptone, 10 g/L NaCl). *S. cerevisiae* MAV203 was used to express recombinant proteins with or without ncAA incorporation; yeasts cells were cultivated in YPD medium (10 g/L yeast extract, 20 g/L peptone, 20 g/L glucose). The recombinant yeast strains expressing enhanced green fluorescent protein (EGFP) and Fab were grown in SC-SCAA medium as previously reported (Wittrup and Benig, 1994). The strains containing aaRS libraries were cultivated, screened, and identified using selective media: SC-Leu-Trp (1.7 g/L YNB, 0.65 g/L SC-Leu-Trp-His-Ura, 5 g/L (NH_4_)_2_SO_4_, 20 mg/L His, 20 mg/L Ura, 20 g/L glucose), SC-Leu-Trp-His-Ura + 3-AT + X-Gal + 10 mM OMeY (1.7 g/L YNB, 0.65 g/L SC-Leu-Trp-His-Ura, 5 g/L (NH_4_)_2_SO_4_, 20 g/L glucose, 10 mM or 30 mM 3-AT, 1% X-Gal, 10 mM OMeY), SC-Leu-Trp-His-Ura + 3-AT + X-Gal and SC-Leu-Trp + TRP + 0.5% 5-FOA.

### Plasmid Construction

Recombinant plasmids were constructed by Gibson assembly (New England Biolabs) using the primers listed in Supplementary Table S1. The gene coding for EGFP-Y39TAG was amplified from plasmid pEGFP-Y39TAG (Tang et al., 2018) and cloned into vector pYX242WS; the resulting plasmid was pYX242WS- EGFP-Y39TAG. The light chain of Fab and the signal peptide *SUC2* from yeast invertase Suc2p were cloned into pYX242WS between promoter *TPI1* and terminator *PGK1*, the recombinant plasmid was named pYX242WS-Lc. The heavy chain of Fab, the signal peptide *SUC2*, and the anchoring motif *SED1* from yeast cell wall protein Sed1p were cloned into pYX242WS-Lc between promoter *TEF1* and terminator *PolyA*, and the recombinant plasmid was named pYX242WS-Fab. Site-directed mutagenesis of pYX242WS-Fab with QuickChange method resulted in pYX242WS-Fab-G123TAG. Plasmids encoding an evolved aaRS/tRNA gene cassette were extracted from library. OMeYRS and its mutant B were constructed into vector pAcBac1, and the recombinant plasmids were named pAcBac1-OmeYRS and pAcBac1-B, respectively. To express recombinant proteins with incorporated ncAA, plasmids with aaRS/tRNA gene cassette and pYX242WS-EGFP-Y39TAG or pYX242WS-Fab-G123TAG were co-transformed into MAV203. HEK293T cells were co-transfected with pCMV-EGFP-Y39TAG and pAcBac1-OMeYRS or pAcBac1-B.

### Library Selection Procedure

Yeast EcTyrRS mutation library for OMeYRS mutant selection was a gift from the Peter Schultz Lab at the Scripps Research. Yeast cells were cultivated in SC-Leu-Trp with 1 mM OMeY for 4 h, harvested and washed twice with 0.9% NaCl buffer. Cell pellets were suspended in 0.9% NaCl buffer, then plated onto SC-Leu-Trp-His-Ura + 10 mM 3-AT + 1% X-Gal + 10 mM OMeY and incubated at 30°C for 48–60 h. To identify OMeY synthetase variants, colonies were transferred into 96 well blocks containing 500 μL of SC-Leu-Trp and cultivated at 30°C for 24 h. Strains were plated onto SC-Leu-Trp-His-Ura + 30 mM 3-AT + 1% X-Gal + 10 mM OMeY, SC-Leu-Trp-His-Ura + 30 mM 3-AT + 1% X-Gal, and SC-Leu-Trp + TRP + 0.5% 5-FOA and grown at 30°C for 48 h.

### Flow Cytometry Analysis

Strains expressing EGFP were cultivated for 24 h, collected and washed twice with phosphate-buffered saline solution (PBS), pH 7.0. Cells were suspended in sterile water to an OD_600_ of 0.5 for fluorescence activating cell sorter (FACS) analysis using a Cytoflex flow cytometer (Beckman). Strains expressing Fab were harvested after 24 h of cultivation and washed twice with PBS. Cell pellets were suspended in PBS containing 1 mg/mL of bovine serum albumin to an OD_600_ of 0.5; then anti-Fab antibody was added to 1:1000 [Alexa Fluor^®^ 647 AffiniPure F(ab’)_2_ Fragment Goat Anti-Human IgG, Jackson 109-606-006]. After incubation at room temperature for 1 h, cells were centrifuged and washed twice with PBS, then suspended in sterile water before FACS analysis.

### Identification of ncAA Incorporation Into Proteins From HEK293

HEK293 cells were cultured in DMEM medium, supplemented with 10% FBS, and incubated at 37°C in 5% CO_2_ to reach 80–90% confluency. Cells were transfected with plasmid pCMV-EGFP- Y39TAG and plasmid pAcBac1-OMeYRS or pAcBac1-B, using PEI reagent (Polysciences). After 4 h of transfection, culture medium was replaced with fresh medium with or without 0.25 mM OMeY for 48 h. Images were taken by immunofluorescence microscopy (Olympus, Japan).

## Results

### Optimization of Screening Scheme for aaRS Selection

**FIGURE 1:**
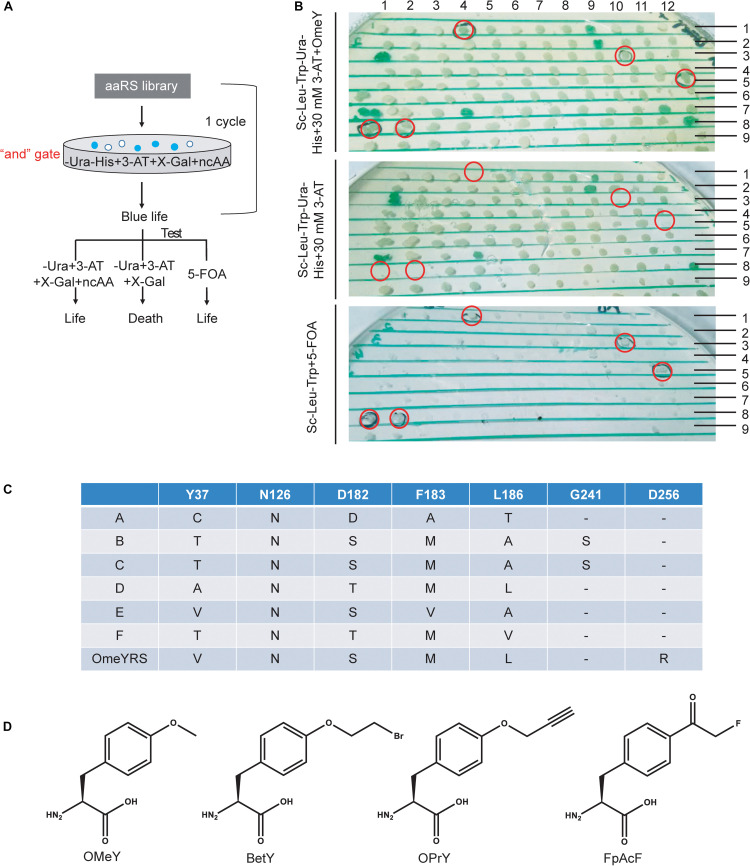
Optimization of a ncAA selection approach *in S. cerevisiae*. **(A)** Diagram of selection scheme and experimental testing in yeast. **(B)** Identification of screened phenotypes of OMeYRS mutants. The presumptive colonies were labeled in red cycles. **(C)** Sequence information of OMeYRS mutants. **(D)** Structure of the ncAAs OMeY, BetY, OPrY, and FpAcF used in this study.

Screening procedures were rationally optimized for selecting specifically active aaRS. In a previous study, an “or” gate was applied for positive selection in the selection scheme (Supplementary Figure S1A), in which cells were cultured without Ura or without His but with 3-AT. Both cultures for the next step selection. Survived cells were collected and mixed to grow in the presence of 5-FOA without ncAA to remove aaRS that could incorporate canonical amino acids. This strategy may generate larger bias and aggravate the screening burden. Here, we changed the “or” gate to an “and” gate, which enriched blue colonies capable of growing in medium without Ura and His by adding target ncAA, 3-AT and X-gal. Cells from blue colonies were tested under three conditions as shown in Figure 1A.

The selection of an OMeYRS mutant from the *Ec*TyrRS mutation library for incorporating OMeY into proteins was used in this study as an example, so new ncAA incorporations can be developed based on this method. The structure of wild-type *Ec*TyrRS is shown in Supplementary Figure S1B. The five sites Y37, N126, D182, F183, and L186 (Supplementary Figure S1B, labeled in red) in the catalytic domain were chosen to perform site-saturation mutagenesis. In the selection procedure, the concentration of 3-AT was optimized to 10 mM. After one round of selection, 192 of blue colonies were picked and identified. As shown in Figure 1B, cells expressing active OMeYRS mutants grew well on media SC-Leu-Trp-Ura-His + 30 mM 3-AT + OMeY and SC-Leu-Trp + 5-FOA, which were used for negative selection. Conversely, these mutants did not grow on SC-Leu-Trp-Ura-His + 30 mM 3-AT. An example of presumptive colony is shown in Figure 1B (first row and the fourth column). We found that approximately 4% of cells were positive, all of which were sequenced and named A, B, C, D, E, F (Figure 1C). The OMeYRS reported previously was used as control (Chin et al., 2003a). However, G241 of B and C mutated to serine, which was not contemplated in the original design. This change could have been caused by random mutation to improve cell adaptation to culture conditions under enhanced selection pressure.

### Incorporation of OMeY Into Recombinant Proteins by OMeYRS Mutants

**FIGURE 2:**
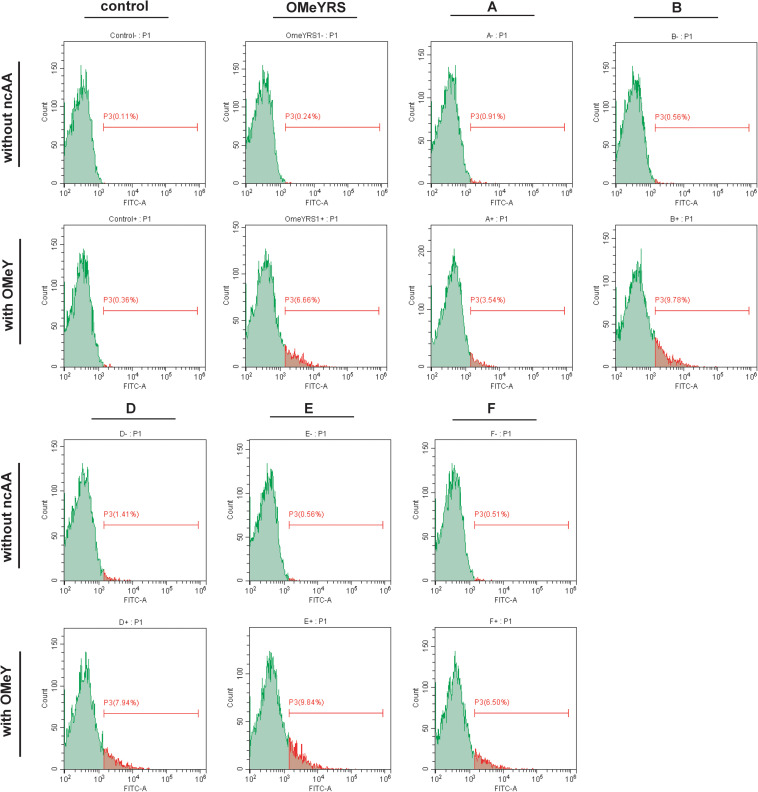
FACS analysis of EGFP with OMeY incorporated by several OMeYRS mutants. Control represents the strain containing empty plasmids. The green peak represented the population of cells that did not express EGFP and had no fluorescence. The control without ncAA was used as background to gate and the population shown in red was gated which expressed EGFP and had fluorescence. The red number represented the proportion of fluorescent cells in total cells. Data analysis was performed using CytExpert Software.

The site-specific insertion of OMeY was verified to demonstrate that the observed phenotypes were due to the five new orthogonal OMeYRS mutants. EGFP response to the UAG condon at position 39 was used as a reporter. As shown in Figure 2, control strain with empty plasmids cultured with or without OMeY did not produce fluorescence signal, whereas the strain expressing OMeYRS showed fluorescence only in the presence of OMeY, revealing that EGFP containing an amber codon could be used for verifying ncAA incorporation. The FACS analysis of the OMeYRS mutant-expressing strains showed that 3.54%(A), 9.78% (B), 7.94% (D), 9.84% (E), and 6.50% (F) of the population positively expressed EGFP by incorporating OMeY, and that EGFP was not expressed in the absence of OMeY (Figure 2). These results showed that several OMeYRS mutants displayed a varied incorporation efficiency, and the strains expressing B, C and E showed a stronger fluorescence signal than that from strains expressing OMeYRS (6.66%), indicating that the expression of EGFP containing OMeY was improved. Thus, the five novel OMeYRS mutants incorporated OMeY and were orthogonal in *S. cerevisiae.*

### Polyspecificity of OMeYRS and Its Mutants

EGFP expressed in cytoplasm and its folding and post-translational modification mechanisms are different for secretion proteins. Antibody libraries are often secreted on cell surface by yeast surface-display platform for antibody-antigen screening. Incorporated ncAA could endow antibodies with new and stable properties (Bidlingmaier and Liu, 2015; Liu et al., 2016). Thus, Heceptin Fab was used as a reporter protein for evaluating aaRS incorporation in *S. cerevisiae*. Yeast cell wall protein Sed1p was selected for anchoring Fab on the yeast cell surface, and the signal peptide from invertase Suc2p was used for driving secretion. Fab was detected using an antibody labeled with Alexa Fluor^®^ 647, so red fluorescent cells were analyzed by FACS. As shown in Supplementary Figure S2, the strain expressing wild type Fab showed a peak shift, whereas both control strain with an empty plasmid and the strain expressing only the light chain of Fab showed a background peak. These results proved that Heceptin Fab was expressed on yeast cell surface, and was a good candidate to characterize the function of aaRS.

**FIGURE 3:**
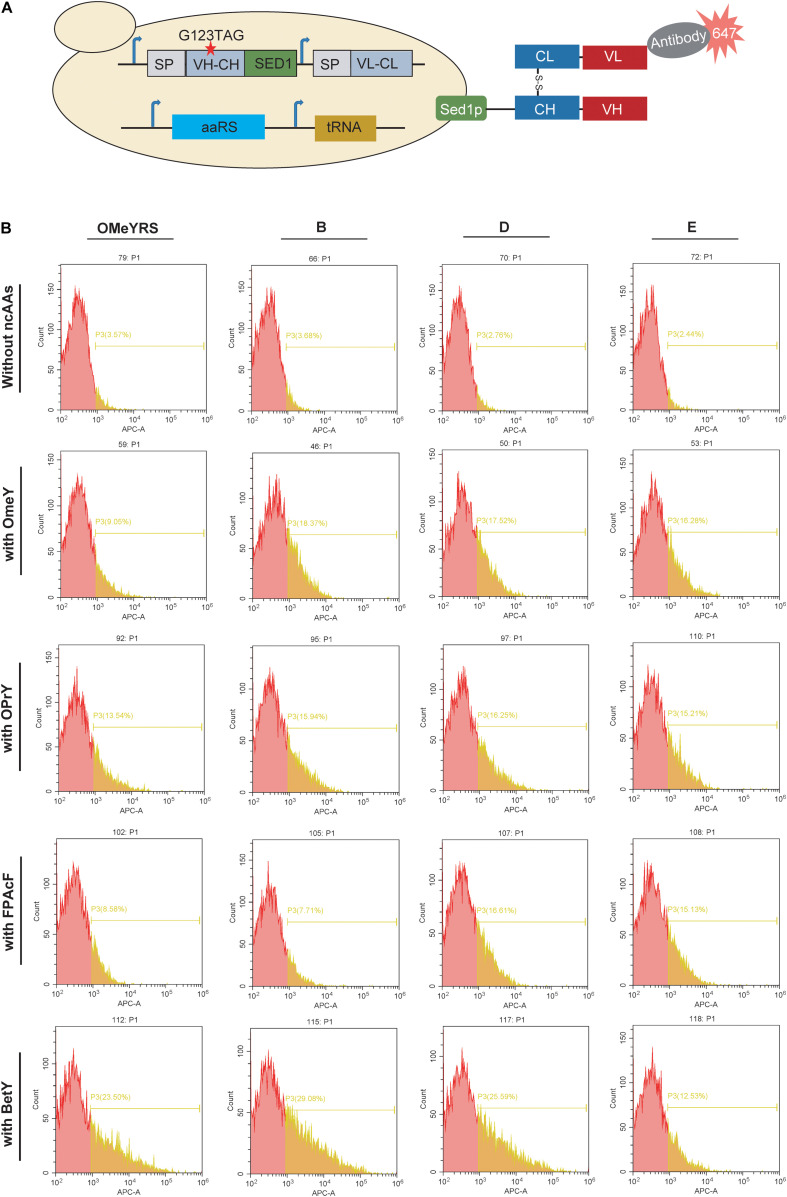
Incorporation of several ncAAs into surface-displayed Fab. **(A)** Schematic representation of surface-display Fab and its detection. SP represented the signal peptide from yeast invertase Suc2p. VH-CH: a variable region of heavy chain (VH) and a constant region of heavy chain (CH); VL-CL: a variable region of light chain (VL) and a constant region of light chain (CL). The red star indicated the mutation site. **(B)** FACS analysis of Fab with ncAAs incorporated by several OMeYRS mutants. Strains expressing Fab were harvested, washed and suspended in PBS containing 1 mg/mL of bovine serum albumin to an OD600 of 0.5, and then anti-Fab antibody was added by 1:1000. After incubation at room temperature for 1 h, cells were centrifuged and washed for FACS analysis. The red peak represented the population of cells that did not stain positively. The yellow number represented the proportion of fluorescent cells in total cells.

Furthermore, the mutant Fab with an amber codon located at complementarity-determining regions was co-expressed with each aaRS. In the presence of ncAA, mutant Fab should anchor on the cell surface as well as wild type Fab, whereas no Fab is present on the cell surface in the absence of ncAA (Figure 3A). As shown in Figure 3B and Supplementary Figure S3, the insertion of OMeY into Fab was achieved by OMeYRS and its mutants. The fluorescence of OMeYRS mutant-expressing strains was higher than that observed in OMeYRS-expressing strains, and the trend was similar in cells expressing EGFP with OMeY. The background fluorescence of Fab-expressing strains was slightly higher than that of EGFP-expressing strains in the absence of ncAA, and this effect could be due to the various properties of different reporters.

To identify the polyspecificity of OMeYRS and its mutants, three additional ncAAs, OPrY, FpAcF, and BetY were tested (Figure 1D), since they can be used for cross-linking reaction (Tang et al., 2018) and bioconjugation (Maza et al., 2016). The FACS analysis showed that the three ncAAs were inserted into Fab by OMeYRS with different incorporation efficiencies. BetY had the highest incorporation efficiency among the three ncAAs, whereas no expression was observed without the addition of ncAA. Strains expressing OMeYRS mutants also showed distinguishable fluorescence peaks upon addition of ncAAs, whereas no sharp peaks were observed without the addition of ncAA, revealing that these mutants charged the three additional ncAAs into Fab instead of canonical amino acids (Figure 3B and Supplementary Figure S3). Except for mutant A, all OMeYRS mutants efficiently inserted OPrY into Fab and 15.94% (B), 16.25% (D), 15.21% (E), and 15.61% (F) of the population stained positively. A (20.79%), D (16.61%), E (15.13%), and F (16.61%) were more suitable for charging FpAcF into Fab compared with OMeYRS (8.58%) and B (7.71%). For the incorporation of BetY, the surface-display efficiency of Fab in strains expressing B (29.08%) and D (25.59%) was about half than that observed in wild type Fab (53.00%, Supplementary Figure S2). These results indicated that OMeYRS and its mutants were polyspecific and were able to charge all four selected ncAAs with minimal background expression in the absence of ncAA. Based on these findings, we concluded that mutant D was the best candidate for inclusion into a single construct.

### Verification of the Function of a New OMeYRS Mutant in Mammalian Cells

**FIGURE 4:**
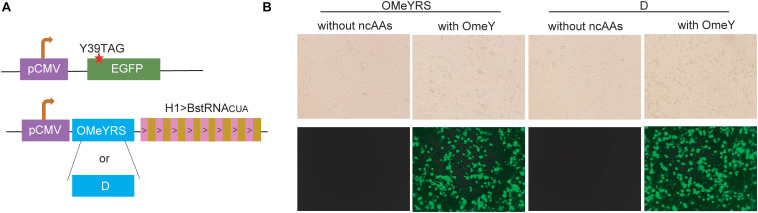
Expression in mammalian cells of EGFP mutants containing OMeY. **(A)** Constructs of *EGFP* and aaRS/tRNA expression cassette. The red star indicated the mutation site. **(B)** Microscopy detection of green fluorescence signal of HEK293 cells transfected with the mammalian expression plasmids. Four hours after transfection, the medium was changed with or without 0.25 mM OmeY, and images were taken at 48 h.

The OMeYRS construct was transfected into mammalian cells, and it worked efficiently with strict orthogonality. Thus, we sought to determine whether OMeYRS mutants could be used for incorporating ncAA into mammalian cells. OMeYRS and mutant D were used as an example and cloned into a plasmid following CMV promoter; the plasmid contained eight copies of *Bacillus stearothermophilus* tRNA^CUA^ driven by H1 promoter. HEK293T cells were co-transfected with a recombinant plasmid containing OMeYRS or mutant D and a plasmid containing EGFP. The amber stop codon was assigned at the position 39 of EGFP (Figure 4A). Bright green fluorescence was detected in both strains expressing OMeYRS and the mutant D in the presence of OMeY (Figure 4B), whereas no fluorescence was observed in the absence of OMeY, suggesting that the expression of full-length EGFP was achieved by both OMeYRS and the mutant D. These results demonstrated that the novel OMeYRS mutants selected by our optimized selection scheme work well in mammalian cells.

## Discussion

*Ec*TyrRS was the first enzyme evolved for site-specific incorporation of tyrosine analogs into proteins in eukaryotic organisms. It has been applied for industrial-scale production of therapeutic antibodies modified by a tyrosine analog due to its high incorporation efficiency (Tian et al., 2014). Even though the selection scheme has been developed in *S. cerevisiae* with practicability and reliability for two decades, only a few structurally simple ncAAs can be incorporated, since the complex and time-consuming screening process remains as one of the limiting factors. To improve the utility of this enzyme by inserting an expanded set of ncAAs with diverse structures, we rationally designed a selection process by using an “and” gate to substitute the commonly used “or” gate. In this strategy, three screening standards including two auxotrophic selection conditions and one blue-white screening method were combined for positive selection, and this selection step is the enrichment process which produce few false positive clones. By adjusting the concentration of 3-AT from 30 to 10 mM, the selection could be accomplished by one round of positive selection and the grown blue clones could be directly picked for verification. Certainly, this positive selection could be combined with negative selection to reduce false positive aaRS, which utilize native amino acids instead of ncAA. The optimized selection scheme used in this study is less time-consuming and simplifies the screening process, which has the potential to be used for rapid aaRS evolution on an extended scale.

The expression of heterologous proteins in *S. cerevisiae* often results in low activity, due to the instability and non-target properties, which limits the production of bioproducts. The introduction of ncAA with specific features could assist protein evolution to produce functional proteins with improved traits, including enhanced stability, high activity, and strong regioselectivity (Jackson et al., 2006; Budisa et al., 2010; Kolev et al., 2014; Ravikumar et al., 2015). For example, the activity of prodrug activator nitroreductase mutants expressed in *E. coli* was improved significantly by incorporating multiple ncAAs, such as *p*-aminophenylalanine, naphthylalanine, *p*-nitrophenylalanine; the latter showed a 30-fold increased activity (Jackson et al., 2006). Thus, an efficient incorporation of several ncAAs would be a desired strategy for enzyme engineering to construct yeast cell factories. In *S. cerevisiae*, the incorporation of lysine analogs by pyrrolysyl-tRNA synthetase/tRNA^CUA^ has been characterized for applications in click chemistry and photocaged and photo-cross-linking reactions (Hancock et al., 2010). Although several structurally simple ncAAs of tyrosine analogs have been incorporated into proteins by *Ec*TyrRS mutants in *S. cerevisiae* (Chin et al., 2003a), their applications are yet to meet the requirements for protein engineering.

To verify the incorporation of several tyrosine analogs in *S. cerevisiae*, we identified the insertion of several ncAAs into recombinant proteins and found that OMeYRS and its mutants screened by a new selection scheme were polyspecific, which was similar to that in mammalian cells (Chatterjee et al., 2013). It is worth noticing that mutant D showed a better performance than OMeYRS for incorporating these ncAAs. Enzyme-substrate docking models suggest that Y37A and F183M mutations increased the binding pocket to accommodate larger sidechains, especially for the *para* position substitution of tyrosine analogs. The F183M mutation, existing in both OMeYRS and mutant D, maintains the hydrophobicity but provides more flexibility to adjust different groups. In addition, the docking results suggest that the substitution groups of OMeY, OPrY, BetY, and FpAcF all pointing toward Y37, which might explain why mutant D (Y37A) outperforms OMeYRS (Y37V) (Supplementary Figure S4). Overall, these results support our data and help to explain the substrate polyspecificity and high incorporation efficiency of mutant D. To the best of our knowledge, this is the first report showing the incorporation of OPrY, BetY, and FpAcF into recombinant proteins in *S. cerevisiae*.

## Conclusion

This study demonstrated that an optimized selection scheme can increase the screening efficiency of aaRS for incorporating specific ncAA into proteins from *S. cerevisiae.* The traditional three-cycle of positive and negative selections was condensed into one round of positive selection, which showed an improved performance and selection efficiency, compared with those obtained by traditional selecting cycles. Several OMeYRS mutants with higher incorporation efficiency were reported using this scheme, among which mutant D was found to be functional after transfection into HEK 293T cells. Additionally, we found that OMeYRS and its mutants are polyspecific to several ncAAs of tyrosine analogs such as OPrY, BetY, and FpAcF. This is the first study addressing the incorporation of OPrY, BetY, and FpAcF into recombinant proteins in *S. cerevisiae*. We consider that this study can be used as a strategy to extend the genetic application of ncAA in *S. cerevisiae* and mammalian cells.

## Data Availability Statement

All datasets generated for this study are included in the article/Supplementary Material.

## Author Contributions

LT, TL, and HT designed the experiments. LT, ZZ, YX, WK, and ZD carried out the experiments. LT, YX, TL, and HT wrote and edited the manuscript. All authors contributed to the article and approved the submitted version.

## Conflict of Interest

The authors declare that the research was conducted in the absence of any commercial or financial relationships that could be construed as a potential conflict of interest.
