# Supplementary material for: Efficient Selection Scheme for Incorporating Noncanonical Amino Acids Into Proteins in Saccharomyces cerevisiae
Source: Front Bioeng Biotechnol. 2020 Sep 15;8:569191. doi: 10.3389/fbioe.2020.569191 (PMC7523088; doi:10.3389/fbioe.2020.569191)
Supplement: Supplementary file 1 [file Table_1.DOCX]

***Supplementary Material***


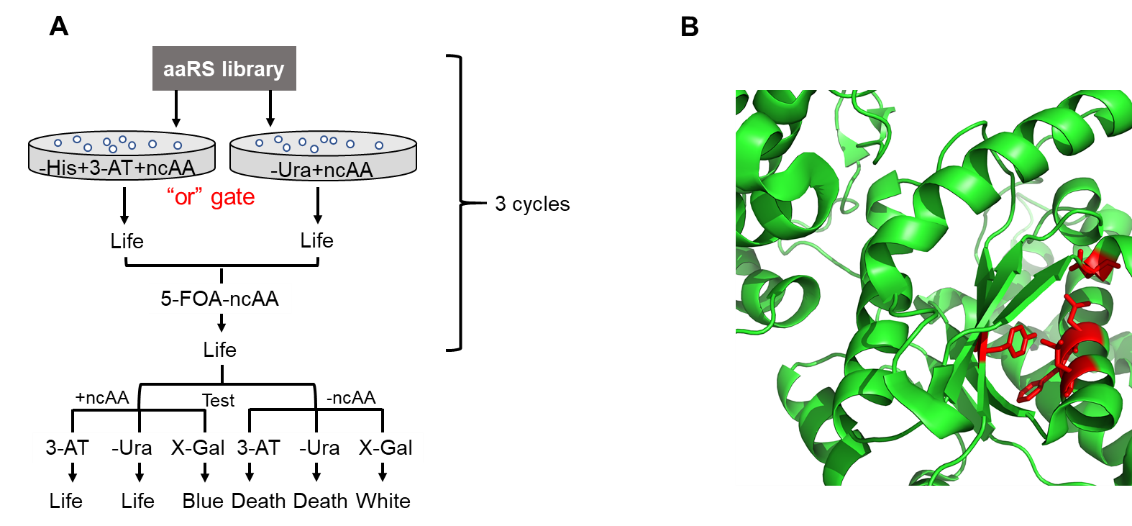


**Supplementary Figure 1** A. General positive and negative selection scheme of aaRS library. B. Crystal structure of EcTyrRS (PDB ID 1X8X). Y37, D182, F183 and L186 are located at catalytic domain and highlighted in red. These residues were performed by saturation mutagenesis to screen efficient OMeYRS for incorporation of OMeY.


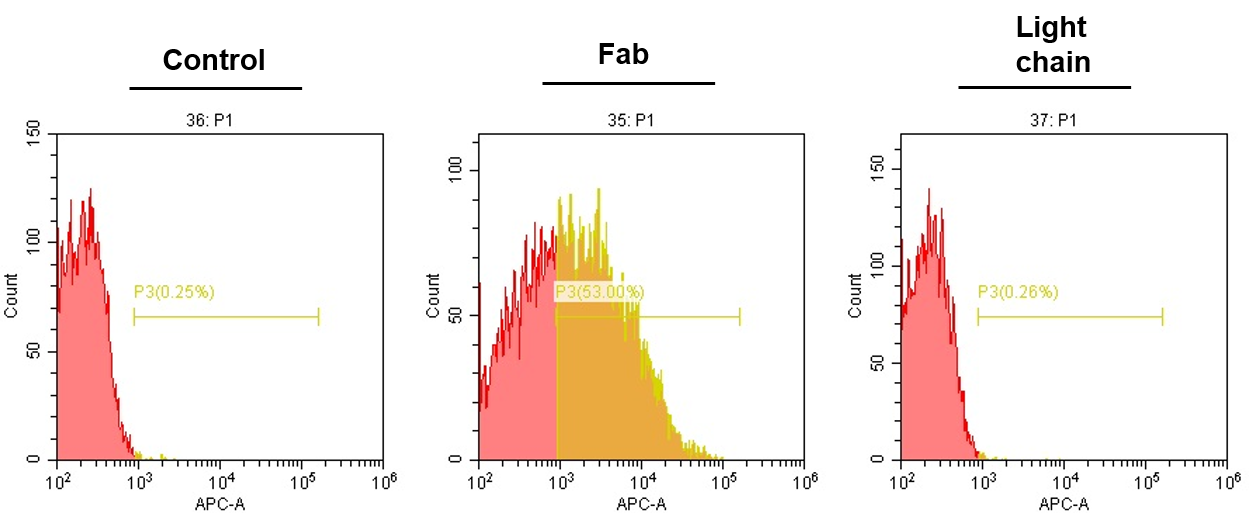


**Supplementary Figure 2** FACS analysis of wide-type Fab on yeast cell surface. Control was the strain with empty plasmid. Light chain was the strain only expressing light chain. Strains expressing Fab were harvested, washed and suspended in PBS containing 1 mg/mL of bovine serum albumin to an OD600 of 0.5, and then anti-Fab antibody was added to 1:1000. After incubation at room temperature for 1 h, cells were centrifuged and washed for FACS analysis. The red peak represented the population of cells that did not stain positively. The yellow number represented the proportion of fluorescent cells in total cells. Data analysis was performed using CytExpert Software.


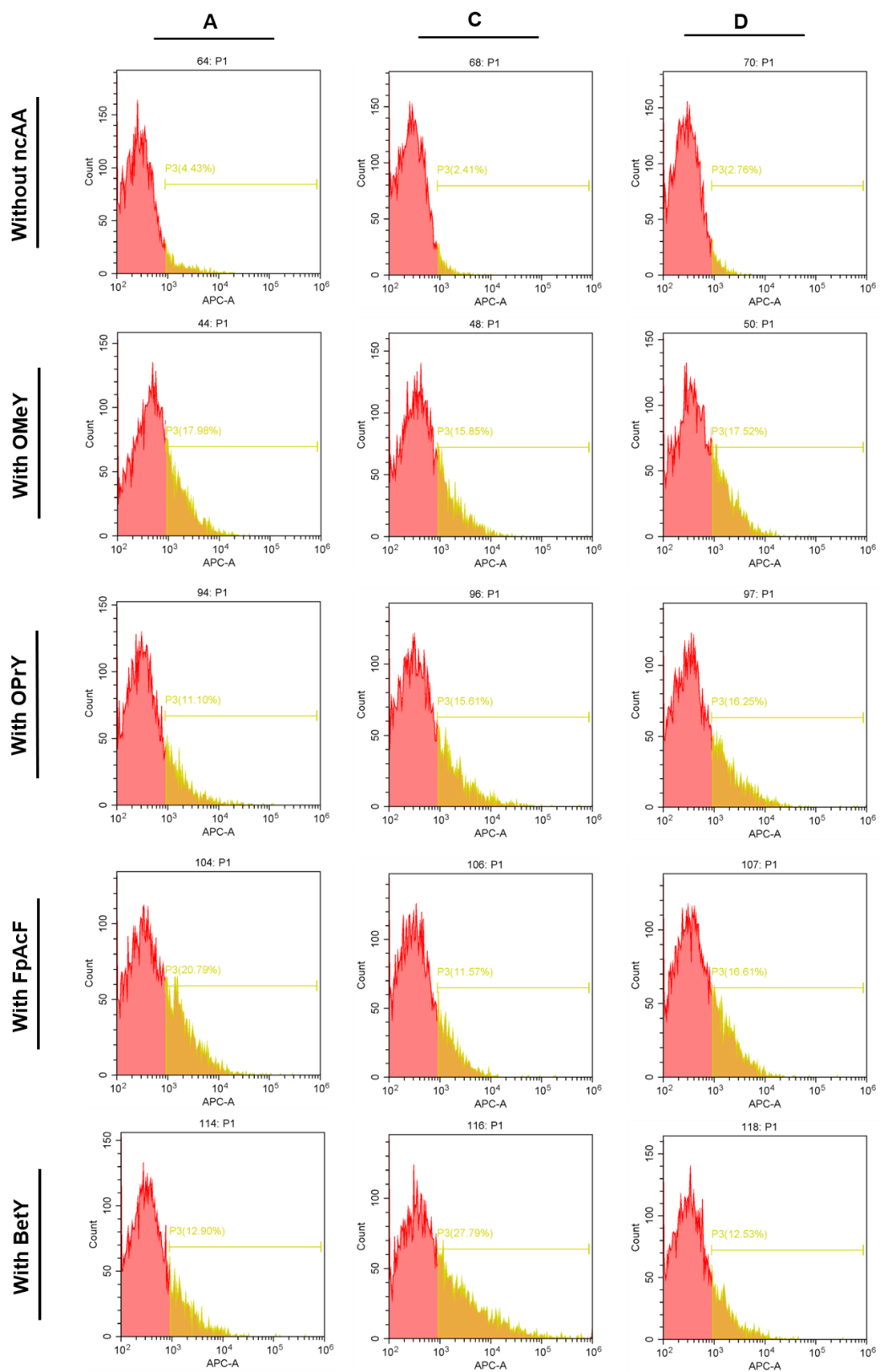


**Supplementary Figure 3** FACS analysis of Fab with incorporation of different ncAAs by OMeYRS mutants A, C, and F on yeast cell surface.


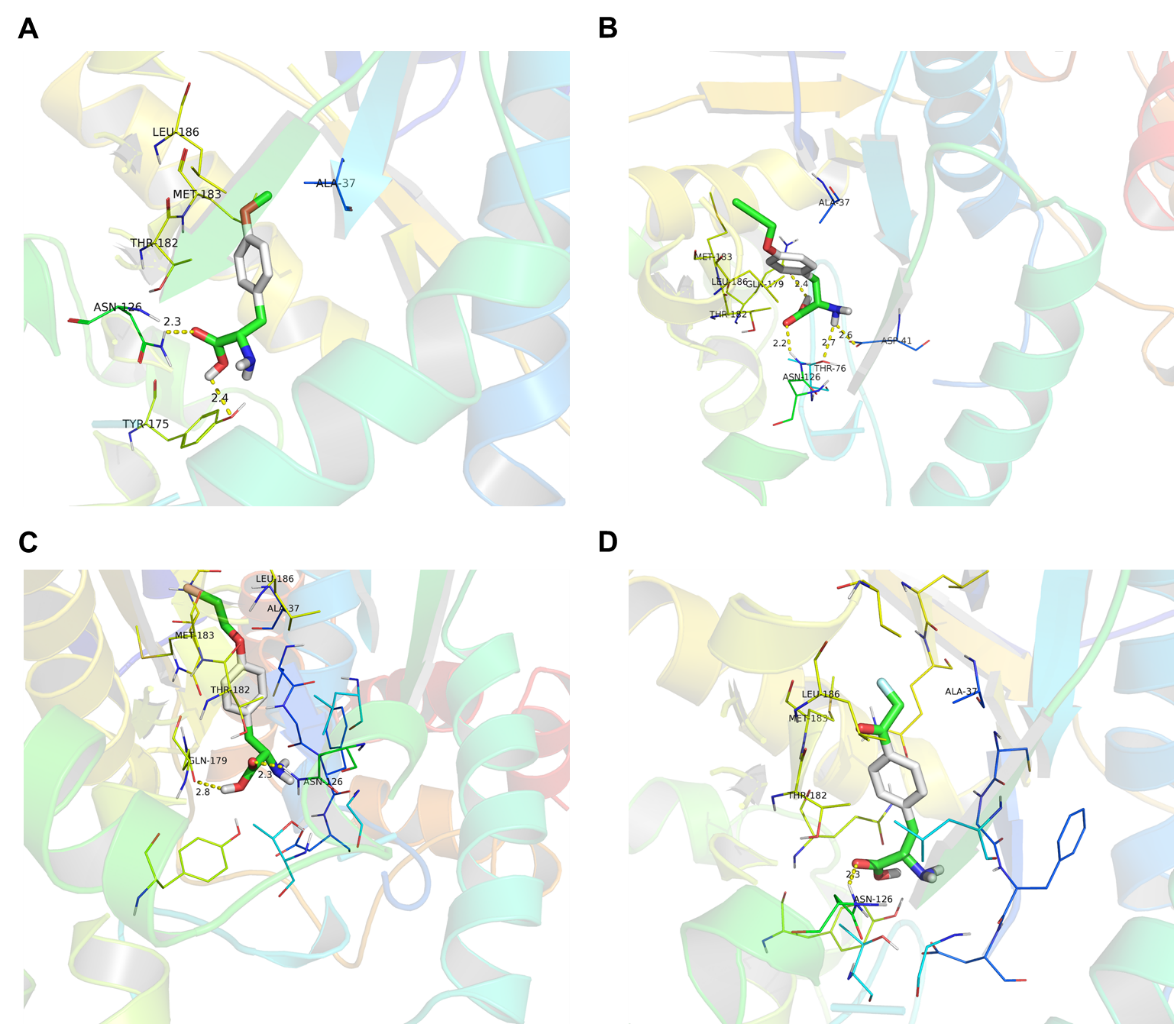


**Supplementary Figure 4** Structural analysis of synthetase D for different ncAAs in: (A) Modeled with OMeY, (B) Modeled with OPrY, (C) Modeled with BetY, (D) modeled with FpAcF. The mutated protein structure was prepared AutoDock-vina (1.1.2), and a series of docking simulations were performed using the implemented Lamarckian genetic algorithm. The cubic energy grid contains the following key sites: A37, T182, M183, L186, N126. The extensions in the X-axis, Y-axis, and Z-axis directions are 41.6Å, 65.0Å, and 48.0Å, respectively. The resulting binding modes were ranked into clusters based on their binding energies.

Table S1 Primes used in this study.

| Name | Sequences (5’-3’) |
| --- | --- |
| pyx-GFP-F | AGCAATCTAATCTAAGTTTTAATTACAAGTCGACATGGTGAGCAAGGGCGAGGAGC |
| pyx-GFP-R1 | CAGTTAGCTAGCTGAGCTCTTAGTGGTGGTGGTGGTGGTGCTTGTACAGCTCGTCCATG |
| pyx-GFP-R | GGAAAAACGTTCATTGTTCCTTATTCAGTTAGCTAGCTGAGCTC |
| Lsuc2-F | CTTAAATCTATAACTACAAAAAACACATACAGGAATTCATGTTGTTGCAGGCTTTCTTG |
| Lsuc-R | GAGACTGGGTCATCTGGATGTCTGATGCAGAAATTTTAGCAGC |
| Lchain-F | GCTGCTAAAATTTCTGCATCAGACATCCAGATGACCCAGTCTC |
| Lchain-R | GAGATCCTAGCTAGCTAGATCCATGGTGAATTCCTAACACTCTCCCCTGTTGAAGCTC |
| Hsuc2-F | ATAGCAATCTAATCTAAGTTTTAATTACAAGTCGACATGTTGTTGCAGGCTTTCTTG |
| Hsuc2-R | GACTCCACCAGCTGCACCTCTGATGCAGAAATTTTAGCAGC |
| Hchain-F | CTGCTAAAATTTCTGCATCAGAGGTGCAGCTGGTGGAGTCTG |
| Hchain-R | AGAACCACCACCACCACTAGCTGTGTGAGTTTTGTCACAAG |
| Sed1-F | CTTGTGACAAAACTCACACAGCTAGTGGTGGTGGTGGTTC |
| Sed1-R | TTGTTCCTTATTCAGTTAGCTAGCTGAGCTCTTATAAGAATAACATAGCAACACCAGC |
| Heavy-tag-F | GTTCGAGATGGGGCGGTGACTAGTTCTATGCCATGGACTACTG |
| Heavy-tag-R | CAGTAGTCCATGGCATAGAACTAGTCACCGCCCCATCTCGAAC |
| 293-RS-F | GACTCACTATAGGGAGACCCAAGCTGGCTAGCGCCACCATGGCAAGCAGTAACTTGAT |
| 293-RS-R | TCCAGAGGTTGATTGTCGACTTAACGCGTTGAATTCTTAAACGGGCCCTTTCCAGC |
| Vector-F | GAATTCAACGCGTTAAGTCGAC |
| Vector-R | GGTGGCGCTAGCCAGCTTGG |
